# The paracaspase MALT1: biological function and potential for therapeutic inhibition

**DOI:** 10.1007/s00018-015-2059-z

**Published:** 2015-10-27

**Authors:** Maike Jaworski, Margot Thome

**Affiliations:** Department of Biochemistry, University of Lausanne, 1066 Epalinges, Switzerland

**Keywords:** NF-κB, Autoimmunity, Lymphoma, Protease, Regulatory T cells

## Abstract

The paracaspase MALT1 has a central role in the activation of lymphocytes and other immune cells including myeloid cells, mast cells and NK cells. MALT1 activity is required not only for the immune response, but also for the development of natural Treg cells that keep the immune response in check. Exaggerated MALT1 activity has been associated with the development of lymphoid malignancies, and recently developed MALT1 inhibitors show promising anti-tumor effects in xenograft models of diffuse large B cell lymphoma. In this review, we provide an overview of the present understanding of MALT1’s function, and discuss possibilities for its therapeutic targeting based on recently developed inhibitors and animal models.

## Introduction

**Fig. 1:**
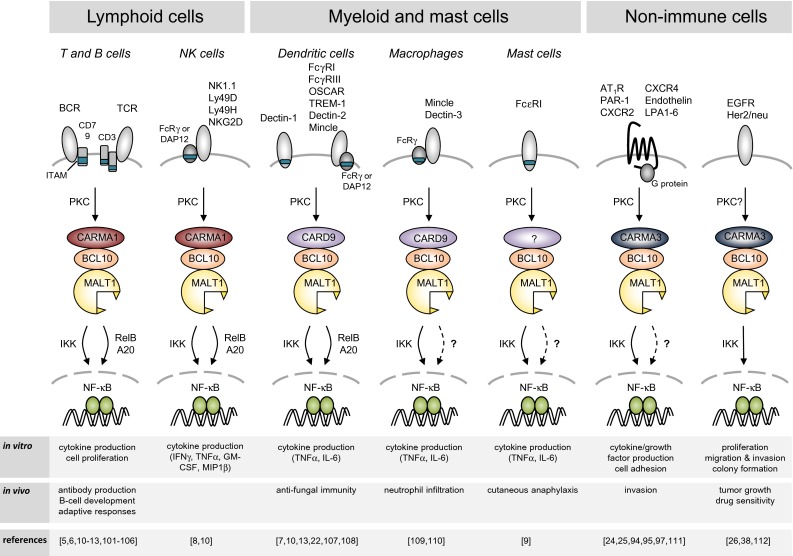
Receptor-induced signaling via MALT1. BCL10 and MALT1 act together to activate NF-κB downstream of receptors containing ITAMs and G protein-coupled receptors. The B- and T-cell receptor (BCR and TCR, respectively) and natural killer (NK)-cell receptors such as NKG2D, NK1.1, Ly49D and Ly49H, mediate signals through CARMA1. ITAM-containing receptors expressed by myeloid and mast cells, such as Dectins, FcγRs, OSCAR, TREM-1 and Mincle mediate signals through CARD9, while GPCRs signal through CARMA3 for the activation of the BCL10-MALT1 module. *ITAM* immunoreceptor tyrosine-based activation motif, *FcRγ* Fc receptor γ-chain; *PKC* protein kinase C, *IKK* inhibitor of NF-κB (IκB) kinase complex, *OSCAR* osteoclast associated, immunoglobulin-like receptor, *TREM-1* triggering receptor expressed by myeloid cells 1

The paracaspase MALT1 plays an essential role in the activation of immune cells by specific subtypes of immune receptors, which induce a common signaling pathway leading to the activation of the transcription factor NF-κB. NF-κB target genes include cytokines and anti-apoptotic proteins, which together promote the activation, proliferation and survival of the activated immune cells upon receptor triggering, and thereby allow the efficient generation of an immune response. MALT1 is also involved in the activation of non-immune cells, in which an NF-κB response can be induced by specific G protein-coupled receptors (GPCRs) or the epidermal growth factor (EGF) receptor (Fig. 1). In this review, we will focus mainly on the role of MALT1 in immune cells. Readers interested in the role of MALT1 in non-immune cells are referred to an excellent recent review covering this topic [1].

## Molecular and biological functions of MALT1

### MALT1 functions downstream of immunoreceptors with an ITAM sequence

The ligand-dependent activation of adaptive and innate immune cells via cell surface receptors that recognize various non-self features is an essential initiating step of the immune response. Not all immune receptors activate MALT1. Indeed, MALT1 specifically transmits signals from immune receptors with a so-called ITAM (immunoreceptor tyrosine-based activation motif), towards the activation of the transcription factor NF-κB (see Fig. 1 and specific references therein). Prototype members of these receptors are the B-cell and T-cell receptor (BCR and TCR), which associate with ITAM-containing CD79 or CD3 chains and recognize antigenic proteins or processed peptide antigens, respectively. Other types of immune cell receptors that signal via ITAM motifs are Fc receptors, which are expressed on myeloid and mast cells and recognize antibody-coated antigenic structures. The myeloid receptors Dectin-1, Mincle and TREM1, contain either an ITAM domain or associate with the ITAM-containing FcRγ chain, and activate innate immune cells upon recognition of microbial glycoproteins [2, 3]. Finally, activating natural killer (NK) cell receptors expressed on NK cells, which associate with ITAM-bearing signaling subunits such as DAP12 or CD3ζ, recognize cellular stress ligands or antigenic structures presented by non-classical MHC molecules [4]. A common consequence of the triggering of ITAM-containing receptors is the NF-κB-dependent expression of proliferation and inflammation-promoting genes, in particular of immune-stimulating cytokines, in the activated immune cells.

### MALT1 has a central role in the immune response

**Fig. 2:**
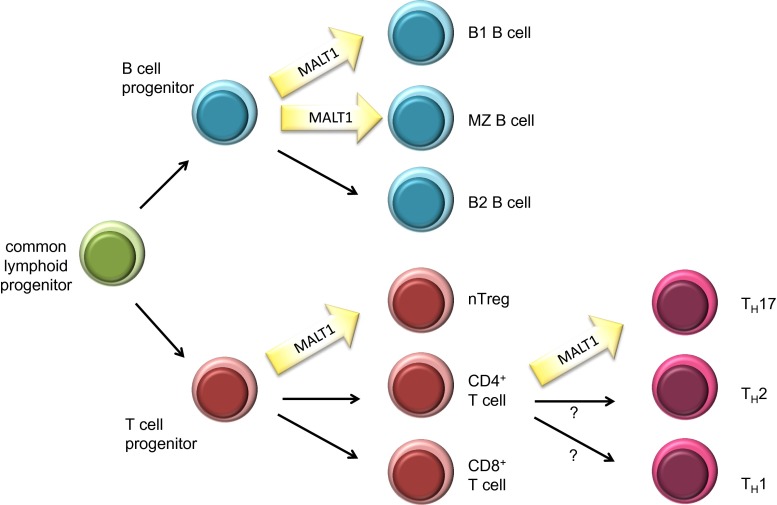
Role of MALT1’s protease function in lymphocyte development and differentiation. MALT1 scaffold and protease functions are essential for the development of peritoneal B1 B cells, marginal zone (MZ) B cells and natural regulatory T cells (nTreg). Polarization of naïve CD4^+^ T cells into the T_H_17 subset of T helper cells is heavily dependent on MALT1 protease function

Genetic studies using MALT1-deficient mice have revealed that MALT1 plays an essential role in immunoreceptor-induced activation events, since mice lacking functional MALT1 are immunodeficient [5, 6]. In particular, these mice show impaired B- and T-cell responses to immunization or viral infection, impaired Fc-receptor mediated cytokine responses of myeloid and mast cells, strongly reduced NK cell responses and impaired innate immunity to yeast infections [5–9]. Additionally, MALT1-deficient mice or mice expressing a catalytically inactive form of MALT1, have impaired development of specific B-cell subsets, such as B1 and marginal zone B cells, and of regulatory T cells [5, 6, 10–13], most likely as a consequence of impaired BCR and TCR signals during lymphocyte development (Fig. 2). MALT1-dependent TCR signaling is also strictly required for the development of effector T cells of the TH17 type [14, 15]. Recently, a small number of human patients with defects in MALT1 expression and/or function have been described [16–18]. A common feature of these patients is combined immunodeficiency, characterized by severe recurrent infections and impaired cellular and humoral immune responses despite normal numbers of circulating B- and T-cells. Collectively, these observations support an essential role for MALT1 in the immune response that is due to its essential signaling function downstream of ITAM-containing immunoreceptors.

### Immunoreceptors with ITAM motifs activate MALT1 via PKC and CARD proteins

**Fig. 3:**
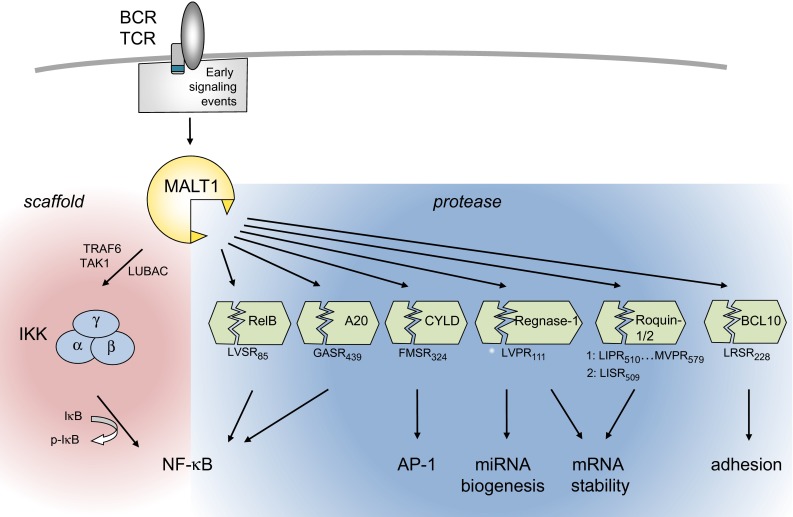
Overview of MALT1-dependent lymphocyte activation via its scaffold and protease functions. Through its scaffold function, MALT1 promotes NF-κB activation via recruitment of the ubiquitin ligase TRAF6, the linear ubiquitin chain assembly complex LUBAC (composed of Sharpin/HOIL/HOIP) and the Ser/Thr kinase TAK1 to activate the IKK complex, which phosphorylates the NF-κB inhibitor IκB to target it for proteasomal degradation. MALT1 protease activity controls NF-κB activation, AP-1 activation, mRNA stability and cellular adhesion by the cleavage of various substrates (see text for details). Positions and sequences of MALT1 cleavage sites in the individual substrates are indicated

How do ITAM-containing immunoreceptors activate MALT1? A common feature of these receptors is that their ITAM motif(s) become(s) rapidly phosphorylated by Src family kinases in response to binding of the antigenic ligand to the receptor. This is followed by the physical recruitment of the Syk family kinases ZAP70 or Syk to the doubly phosphorylated ITAM motif [19]. The signal is then relayed by Ser/Thr kinases of the PKC family, which in turn phosphorylate the scaffold protein CARMA1 (CARD-MAGUK1, also known as CARD11) [20, 21] or the CARMA1 homologue CARD9 [22], thereby promoting the physical assembly of oligomeric CARMA1/CARD9-BCL10-MALT1 (CBM) complexes (Fig. 1). The stoichiometry of these complexes is not well defined; recent findings suggest that the CBM signalosome is a filamentous assembly in which CARMA1 nucleates formation of BCL10 filaments that are decorated with MALT1 [23]. In non-immune cells, the CARMA1 homologue CARMA3 (also known as CARD10) is thought to play a similar role in promoting BCL10- and MALT1-dependent NF-κB activation downstream of G protein-coupled receptors or the EGFR [24–26]. The CARMA1-homologue CARMA2 (also known as CARD14) has been proposed to drive NF-κB activation in keratinocytes of the skin [27, 28], but the upstream signals triggering CARMA2 activation remain unknown. As a consequence of its BCL10-dependent physical recruitment into a CBM complex, MALT1 promotes NF-κB activation via both its scaffold and protease functions (Fig. 3). As a scaffold, MALT1 recruits ubiquitin ligases that promote the activation of the IκB kinase (IKK) complex [29–33]. IKK-dependent phosphorylation targets the NF-κB inhibitor, IκB, for proteasomal degradation and thereby allows NF-κB subunits to enter the nucleus and to initiate the transcription of NF-κB target genes [34]. The protease activity of MALT1, on the other hand is essential for NF-κB signal amplification and persistence, which is achieved mainly by the cleavage of two negative regulators of the NF-κB pathway, namely RelB and A20 [35, 36]. So far, the relevance of MALT1 protease activity for NF-κB activation has been formally demonstrated for receptors depending on CARMA1 or CARD9 [10–13, 35, 37]; whether it is also relevant for CARMA2- and CARMA3-dependent NF-κB signals remains uncertain [38]. Recently, several new substrates of MALT1 have been identified. These substrates control additional aspects of leukocyte activation such as activation of AP-1 transcription factors and regulation of transcript stability [39–41]. The molecular features of the MALT1 scaffold and protease functions and their biological relevance are further detailed below.

### The scaffold function of MALT1 is defined by its protein binding motifs

**Fig. 4:**
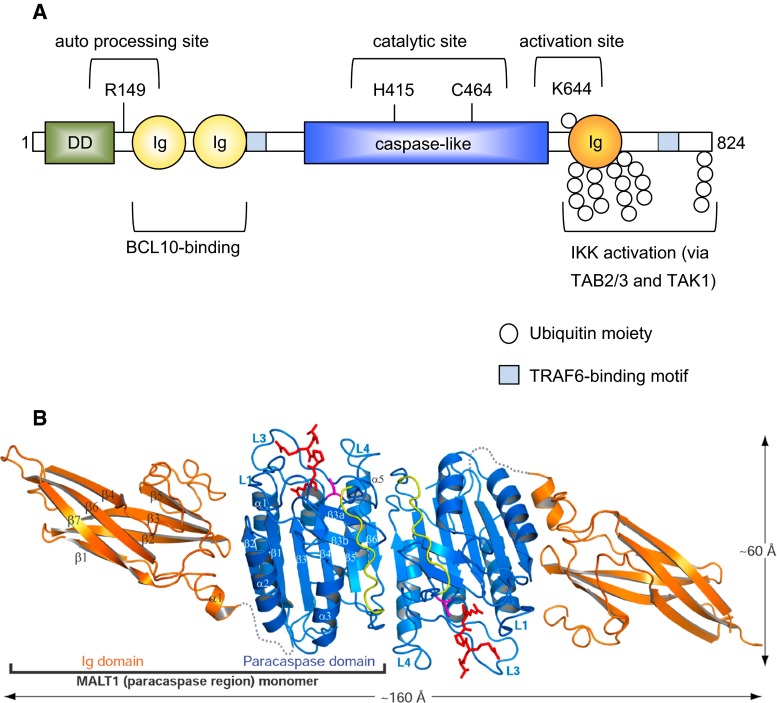
Molecular structure and function of MALT1. **a** MALT1 contains an N-terminal death domain (DD), followed by two immunoglobulin-like domains (Ig), which are required for the interaction between MALT1 and BCL10. The central caspase-like domain, with the active site residues H415 and C464, is followed by a third Ig domain that contains K644, a monoubiquitination site that controls protease activity. MALT1 contains two binding motifs for the ubiquitin ligase TRAF6 (tumor necrosis factor receptor-associated factor 6). TRAF6 polyubiquitinates MALT1 on multiple C-terminal lysine residues, generating K63-linked ubiquitin chains that can in turn promote activation of the inhibitor of NF-κB kinase (IKK) complex through recruitment of the IKK-activating kinase TAK1 via the adaptor proteins TAB 2/3. Amino acid numbering in the figure refers to human MALT1. **b** Ribbon representation of the crystallographic structure of the MALT1 homodimer (figure reproduced from Yu et al., PNAS 108 (52), 21004–21009 (2011), with kind permission) [45]. The shown crystallized fragment comprises the paracaspase region (*blue*) and the adjacent C terminal Ig domain (*orange*). The covalently bound MALT1 peptide inhibitor is colored *red* and the subunit linker in the caspase-like fold is colored *yellow*. The sequences between the caspase-like domain and the Ig domain are disordered and represented as *gray* *dotted lines*

Over the last few years, our understanding of the function of MALT1 has considerably been improved by molecular and biochemical analyses, which have dissected individual functions of specific structural subdomains present in the MALT1 protein (Fig. 4a). MALT1 contains a predicted N-terminal death domain of unknown function, followed by two immunoglobulin (Ig)-like domains that have been shown to be essential for its constitutive binding to the adaptor protein BCL10 [42, 43]. In addition, MALT1 contains a central catalytic domain that has homology with proteases of the caspase family [42]. Shared features include a conserved Cys and His residue required for catalysis and a typical three-dimensional fold of the protease domain, which can dimerize [42, 44, 45] (Fig. 4b). The protease domain is followed by a third Ig-like domain that contains important sites for mono- and polyubiquitination, and by a C-terminal extension of unknown structure. The capacity of MALT1 to recruit the ubiquitin ligase TRAF6 and to thereby activate the IKK complex has been attributed to two individual TRAF6 binding motifs located outside the protease domain [30, 31] (Fig. 4a). TRAF6 recruitment to MALT1 is thought to promote IKK activation by polyubiquitination of MALT1 together with its binding partner BCL10 and TRAF6 itself [29, 31, 46]. The resulting K63-linked polyubiquitin chains are thought to provide docking sites for the recruitment of the kinase TAK1 via TAB 2/3 adaptor proteins [47, 48], which allows TAK1 to phosphorylate and thereby activate the catalytic IKK subunit IKKβ [31, 48]. MALT1 also associates with the linear ubiquitination chain assembly complex LUBAC (also known as the Sharpin/HOIL/HOIP complex) [32, 33], but the consequences of this interaction for IKK activation remain incompletely understood. It is most likely that the linear ubiquitination of CBM- or associated components provides docking sites for the physical recruitment of the IKK complex through the linear ubiquitin chain-binding UBAN motif of the IKK subunit IKKγ (also known as NEMO) [47, 49]. Collectively, these findings support the idea that MALT1 can act as a scaffold to coordinate the recruitment and activation of TRAF6 and the kinases TAK1 and IKK for NF-κB activation.

### The protease activity of MALT1 is required for optimal NF-κB and AP-1 activation

Over the last years, it has become clear that MALT1 signaling downstream of ITAM-containing receptors depends not only on its scaffold function, but to a large extent on its protease activity. A specific feature of MALT1 activation that differentiates it from proteases of the caspase family is the requirement of the third Ig domain (Ig3), which forms several hydrophobic contacts with the adjacent protease domain that are released upon substrate binding (Fig. 3a, b) [44, 45]. This suggests a model in which the auto-inhibitory Ig3 domain contributes to MALT1 activation by an inducible conformational change in this region [44, 50]. Interestingly, MALT1 activity is tightly controlled by antigen receptor-induced monoubiquitination of MALT1 on residue K644 within the Ig3 domain (Fig. 3a), and this modification induces or stabilizes MALT1 dimerization via the protease domain [51, 52].

With the discovery of the proteolytic activity of MALT1 and of specific MALT1 protein substrates, our understanding of MALT1 function has been considerably enriched (Fig. 3c). The protease activity of MALT1 is thought to promote NF-κB activation through the cleavage of the deubiquitinating enzyme A20 and of the NF-κB subunit RelB. How exactly A20 cleavage affects NF-κB activation remains unclear. A20 can negatively regulate IKK-dependent NF-κB activation by deubiquitination of MALT1 [53], and A20 levels decrease shortly after T-cell stimulation [53]. However, MALT1 activity is neither required for A20 degradation [53] nor for IKK-dependent IκBα phosphorylation [10–12, 36, 53], and the proportion of inducible A20 cleavage is relatively small [35]. This suggests that MALT1-dependent A20 cleavage may serve to generate an A20 fragment that affects NF-κB activation in an IKK-independent manner that remains to be further explored. RelB, which is mainly known for its activating role in the alternative NF-κB pathway, acts as a negative regulator of canonical NF-κB activation by the antigen receptor [36, 54–56], most likely through the formation of transcriptionally inactive RelA/RelB heterodimers and/or competition at the DNA binding site [36, 57, 58]. MALT1-dependent RelB cleavage results in its proteasomal degradation, thereby lowering total levels of RelB in activated B- and T cells [36]. Through cleavage of RelB and A20, MALT1 thus expands the amplitude and duration of the NF-κB response in an IKK-independent manner. Interestingly, MALT1 also promotes NF-κB activation by its autoprocessing after Arg 149. The exact consequences of MALT1 autoprocessing remain unclear, but include an effect on NF-κB target gene expression downstream of nuclear NF-κB accumulation [59].

In addition to having defects in the NF-κB pathway, MALT1-deficient cells were also reported to have an impaired capacity to activate the JNK/AP-1 transcriptional pathway [5]. This may be attributed at least in part to the requirement of MALT1 for the cleavage of the deubiquitinating enzyme CYLD [39], which negatively regulates this pathway in lymphocytes through deubiquitination of the JNK upstream kinase TAK1 [60]. Expression of a non-cleavable form of CYLD inhibited activation of the JNK pathway and expression of AP-1 target genes in a T-cell line, although CYLD silencing only minimally increased JNK activation [39], possibly because of redundancy with other deubiquitinating enzymes. Of note, recent studies with protease-inactive MALT1 knock-in mice have shown that MALT1 protease activity is not required for JNK activation [10–12]. Thus, the exact mechanism of MALT1- and CYLD-dependent AP-1 activation and the role of JNK in this pathway remain to be further explored.

### MALT1 controls transcript stability by cleavage of mRNA-destabilizing proteins

An entirely new aspect of MALT1’s biological function has been revealed with the recent discovery that Regnase-1 (also known as MCPIP1 or Zc3h12a) and the Roquin-1 and -2 proteins act as MALT1 substrates [40, 41]. Regnase-1 is an RNAse with an important function in mRNA degradation. Regnase-1 binds to specific mRNA stem loop structures and promotes mRNA degradation by its RNAse activity [61] and by recruitment of the RNA remodeling helicase UPF1 [62]. It was additionally suggested that Regnase-1 controls miRNA generation by processing of premiRNAs [63]. MALT1-dependent cleavage of Regnase-1 lowers its levels in activated T cells and thereby induces stabilization of a subset of pro-inflammatory transcripts, such as IL-6, IL-2, c-Rel and ICOS [40, 41]. As a consequence, MALT1-dependent Regnase-1 cleavage promotes T-cell activation [40]. Mice with a T-cell specific Regnase-1 deficiency have exaggerated T-cell responses, which account, to a large part, for the reported autoimmunity and systemic inflammation features of Regnase-1-deficient mice [61]. The MALT1 substrates Roquin-1 and Roquin-2 promote mRNA degradation by binding to 3’ located stem-loop structures in mRNA molecules known as conserved decay elements (CDE) [64, 65]. This allows Roquin proteins to promote mRNA deadenylation by recruitment of the cellular CCR4-CAF1-NOT deadenylase complex [64]. MALT1-dependent cleavage of Roquin proteins has been proposed to play a predominant role in the stabilization of specific transcripts, such as IL-6, ICOS, c-Rel and IRF4, which are also targets of Regnase-1 and collectively promote the differentiation of T effector cells of the TH17 subtype [41]. In addition to its roles in transcription and transcript stabilization, MALT1 has been suggested to have a role in promoting T-cell adhesion through cleavage of its binding partner BCL10, by mechanisms that are not yet understood [37]. Collectively, these findings suggest a broad role for MALT1, and in particular for its protease activity, in immune receptor-induced activation of transcription, RNA stabilization and cellular adhesion (Fig. 3).

### Additional, less explored roles for MALT1 and its protease activity in lymphocytes

Beyond its established roles in activation of NF-κB and AP-1 and transcript regulation, MALT1 most likely controls other cellular activation pathways. It has for example been proposed that MALT1 controls NF-κB2 activation downstream of the BAFF-receptor in specific B-cell subsets, possibly via receptor-induced dissociation of MALT1 from the ubiquitin ligase TRAF3 [66] that negatively regulates BAFF-receptor-dependent survival signals [67]. Recently, MALT1 protease activity has been shown to be required for TCR/CD28-dependent induction of glutamine uptake and mTOR activation that controls metabolic changes required for T-cell activation [68]. The substrate responsible for this effect remains unknown.

## MALT1 inhibition and its therapeutic potential for lymphomas

### Different compounds can be used to inhibit MALT1 in vitro and in vivo

MALT1’s enzymatic activity has stimulated efforts to develop specific MALT1 inhibitors for research and therapeutic purposes. The first MALT1 inhibitor identified was z-VRPR-fmk, a modified tetrapeptide based on the optimal substrate Val-Arg-Pro-Arg of the *Arabidopsis thaliana* metacaspase AtmC9, conjugated to fluoromethyl ketone (fmk) [37]. This modified peptide irreversibly blocked MALT1 protease activity in an in vitro cleavage assay using recombinant MALT1 in a dose-dependent manner. It furthermore efficiently inhibited T cell activation and IL-2 secretion in Jurkat T cells and in human antigen specific CTLs [37]. An alternative version of this inhibitor, named z-LVSR-fmk, which is based on the LVSR substrate sequence in the MALT1 substrate RelB [36], also inhibits MALT1 efficiently [59]. Recently, two types of potential small molecule MALT1 inhibitors have been identified in high throughput screening approaches [69, 70], suggesting that it will be feasible to develop suitable MALT1 inhibitors for in vivo studies. Nagel and colleagues have identified three phenothiazine derivatives (mepazine, thioridazine, and promazine) as highly specific, noncompetitive and reversible MALT1 inhibitors [69]. A concurrent study by Fontan and colleagues has identified the compound MI-2 as a selective MALT1 inhibitor [70]. In contrast to phenothiazine derivatives, MI-2 engages and irreversibly binds the active site of MALT1 [70]. Co-crystallization of thioridazine with MALT1 has revealed that these compounds bind the interface between the protease domain and the Ig3 domain of MALT1, an allosteric site that is far from the active site of the enzyme [71]. Thus, thioridazine most likely affects MALT1 activity by preventing a conformational change in the protease-Ig3 interface that is essential for MALT1 activation [44, 45, 50]. Recently, two studies have reported the first generation of MALT1 activity-based probes derived from peptide- or phenothiazine inhibitors [72, 73]. While these are yet of limited sensitivity, improved probes may become useful in the future to detect MALT1 activity in pathological settings or for measuring patient responses to MALT1 inhibitor treatment.

**Fig. 5:**
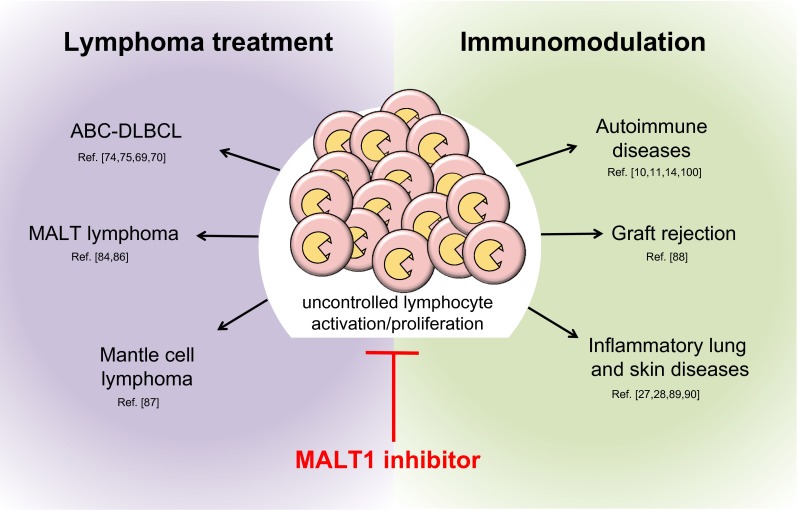
Potential fields of application of clinical MALT1 inhibitors. Possible applications include treatment of lymphomas with constitutive MALT1 activity and immunomodulation in the context of transplantation tolerance, autoimmunity and various inflammatory disorders

The potential applications of MALT1 inhibitors in the fields of immunomodulation and the treatment of lymphomas are reviewed below and illustrated in Fig. 5.

### MALT1 inhibition could be a strategy to target ABC DLBCL lymphomas

The first indication that Malt1 inhibition might be a promising strategy to treat human diseases came from two studies in 2009 which reported a preferential cytotoxicity of the MALT1 inhibitor z-VRPR-fmk on a subtype of cell lines derived from diffuse large B-cell lymphoma (DLBCL) [74, 75]. DLBCL can be genetically classified into molecularly distinct subtypes, including the germinal center B cell (GCB) and the activated B cell (ABC) subtype. Human cases of GCB DLBCL generally show a slow and chronic progression of the disease, whereas cases of ABC DLBCL have a faster course, worse 5 year survival rate and respond less well to chemotherapeutic treatment. Growth of ABC DLBCL is driven by constitutive NF-κB signaling that results from a variety of mechanisms [76]. These include activating mutations in the BCR-associated CD79A/B chains, (present in roughly 23 % of ABC DLBCL cases [77, 78]) the MALT1 activator CARMA1 (roughly 8 % of cases [77, 79]) or the TLR adaptor protein MyD88 (37 % of cases) [80], together with inactivating mutations in A20, a negative regulator of the NF-κB pathway (23 % of cases) [81]. Using an RNAi screen on ABC DLBCL cell lines, Ngo and colleagues demonstrated that these cell lines, which have combined mutations in MyD88 and either the CD79 or CARMA1 proteins, heavily depend on NF-κB activation via CARMA1, BCL10 and MALT1 for their survival and proliferation [82]. Inhibition of MALT1 by treatment with z-VRPR-fmk, or by expression of a catalytically inactive form of MALT1, decreased the expression of NF-κB target genes and dramatically reduced the viability and growth of cell lines derived from ABC DLBCL, but not from GCB DLBCL [74, 75]. Collectively, these findings suggested a key role for MALT1 activity in the growth of ABC DLBCL cells.

Recently, small molecule inhibitors of MALT1 have been shown to be effective against ABC DLBCL in xenograft models [69, 70]. The phenothiazine derivatives identified by Nagel et al., which act as allosteric MALT1 inhibitors, showed selective activity against ABC DLBCL cell lines in vitro and xenotransplanted ABC DLBCL tumors in vivo [69]. The compound MI-2, an active site MALT1 inhibitor developed by Fontan et al., also successfully suppressed the growth of human ABC DLBCL xenografts in mice without notable toxicity [70]. Thus, MALT1 inhibitors may have potential in the treatment of ABC DLBCL, in particular for those cases that have oncogenic CARMA1 mutations that will render them insensitive to compounds inhibiting BCR signaling upstream of CARMA1, such as the Btk inhibitor Ibrutinib [76].

### Additional types of lymphomas may be targeted by MALT1 inhibitors

Another type of lymphoma with constitutive MALT1 activity is the MALT lymphoma. A large proportion of cases with advanced stages of this disease are characterized by the presence of a chromosomal translocation (t(11;18)(q21;q21) that encodes the oncogenic IAP2-MALT1 fusion protein, composed of the N terminus of IAP2 linked to the C terminus of MALT1. The IAP2 moiety mediates auto-oligomerization of the fusion protein [83], rendering IAP2-MALT1 constitutively active and able to stimulate NF-κB independently of upstream signals. The IAP2–MALT1 fusion recruits the ubiquitin ligase TRAF6 to promote canonical, IKK-dependent NF-κB1 activation [30]. In addition, it has constitutive protease activity as a consequence of its monoubiquitination [51], and can cleave natural MALT1 substrates such as A20 to promote NF-κB1 activation [35]. Aside from activating the canonical NF-κB pathway, the IAP2–MALT1 fusion protein specifically triggers NF-κB2 activation by the cleavage of the Ser/Thr kinase NIK, which is detectable in MALT lymphoma samples [84]. NIK promotes the activation of the NF-κB2 pathway through phosphorylation and subsequent proteolytic maturation of the NF-κB2 precursor p100 into its transcriptionally active form p52 [85]. Normally, the cellular activity of NIK is limited by its very short half-life, but MALT1-dependent cleavage of NIK generates a NIK fragment that is stable and active, thereby promoting constitutive NF-κB2 activation to drive cellular adhesion and protection from apoptosis of the malignant cells [84]. In addition to cleaving NIK, the IAP2-MALT1 fusion protein has recently been shown to cleave the tumor suppressor protein LIMA1, and LIMA1 cleavage products are present in MALT lymphoma samples expressing IAP2-MALT1 [86]. LIMA1 cleavage abolishes its tumor suppressor function and generates a novel fragment with oncogenic properties that contributes to cellular transformation [86]. The IAP2-MALT1 fusion protein thus seems to drive cellular transformation by multiple means, suggesting that inhibition of the MALT1 protease activity could be an attractive new treatment approach for t(11;18)-positive MALT lymphoma.

Finally, a recent study by Rahal and collegues [87] identified a subset of MCL lines with chronic activation of the BCR mediated CBM–NF-κB signaling pathway and constitutive RelB cleavage, suggesting that a subset of MCL may also be responsive to MALT1 inhibition. While MALT1 activity has not yet been assessed in other lymphoma types, it seems likely that additional types of B- or T-cell lymphomas with constitutive antigen receptor signaling are sensitive to MALT1 inhibition.

## Use of MALT1 inhibition for immunomodulation

### MALT1 may control transplant rejection

The potential relevance of MALT1-dependent signaling has thus far only been addressed using mice deficient for the MALT1 upstream regulator CARMA1 and pancreatic islet allografts [88]. Transplanted CARMA1-deficient mice displayed a lack of T cell priming, showed very few mononuclear cell infiltrates in the grafts and accepted fully allogeneic islet allografts long-term. These results hold promise that MALT1 inhibition might be beneficial in tissue transplantation, especially for the transplantation of less immunogenic tissues. However, since MALT1 protease inhibition is less efficient in preventing T cell activation than complete CARMA1-deficiency, the benefits will have to be determined experimentally. In addition, it is of concern that CBM deficient mice as well as mice expressing a catalytically inactive form of MALT1 display a developmental lack of regulatory T (Treg) cells. Considering that Treg cells play a critical role in preventing graft rejection [15], this lack of Treg cells will complicate the interpretation of grafting experiments using these mouse models. Whether prolonged treatment of mice with a MALT1 inhibitor could adversely affect engraftment by reducing Treg numbers remains to be determined.

### MALT1 may be relevant for inflammatory lung and skin disorders

CARMA1 has been shown to play an important role in the induction of allergic asthma [89]. Immunization and challenge of wild type mice with an allergen leads to eosinophilic airway inflammation, with production of Th2 cytokines and IgE, mucus cell hypertrophy and airway hyperresponsiveness, whilst CARMA1-deficient mice are protected from disease induction. A follow up study by the same group [90] revealed that CARMA1 is also important in recall responses when the initial priming has occurred in the presence of CARMA1, which more closely resembles the natural conditions of a therapeutic intervention. The CARMA1 homologue CARMA3 (also known as CARD10) that is expressed by airway epithelial cells is implicated in allergic airway inflammation by mediating proinflammatory cytokine production, DC maturation and migration, antigen processing and T cell proliferation [91]. MALT1, which acts downstream of both CARMA1 and CARMA3, may thus bridge innate and adaptive immune responses in the context of allergic asthma. Interestingly, mutations in the CARMA1 homologue CARMA2 (also known as CARD14) have been recently associated with the development of psoriasis in human patients [27, 28]. Some of these mutants were found to be hyperactive and to promote expression of psoriasis-associated chemokines such as CCL20 and IL-8 in keratinocytes [28]. Collectively, these findings suggest that inhibition of CARMA1/2/3-dependent signaling with a MALT1 inhibitor may have potential in the treatment of various inflammatory disorders through effects on immune and non-immune cells.

### MALT1 may play an important role in GPCR- and EGFR-driven pathologies

To date, little is known about the requirement of MALT1 protease activity for signaling by GPCR or the EGFR, which both depend on MALT1 and the upstream regulator CARMA3 (also known as CARD10) for NF-κB signaling (see Fig. 1 and references therein). EGFR signaling does not appear to depend on MALT1 protease activity [38], while the requirement for MALT1 protease activity for signaling by GPCRs has not yet been thoroughly investigated. Signaling by GPCRs depending on CARMA3 and MALT1 regulates various inflammatory processes, including lysophosphatidic acid (LPA)-induced lung inflammation [92], platelet-activating factor (PAF)-induced intestinal inflammation [93] and angiotensin II-, thrombin- and IL-8-mediated vascular inflammation [24, 94, 95]. Moreover, GPCR-signaling via CARMA3 and MALT1 may be important for LPA- or SDF-1-induced carcinogenesis [96, 97]. Thus, whether MALT1 inhibition might positively affect these pathological conditions merits further investigation.

### MALT1 protease-deficient mice reveal contradictory roles for MALT1 in autoimmunity

To assess the biological relevance of the MALT1 protease activity in vivo, several groups have recently generated mice expressing a catalytically inactive form of the MALT1 protease (MALT1 protease dead, MALT1-PD) [10–13]. CARMA1- or MALT1-deficient mice are fully protected from induction of experimental autoimmune encephalomyelitis (EAE) by immunization with myelin oligodendrocyte glycoprotein (MOG) [14, 98, 99], and treatment of mice with the MALT1 inhibitor mepazine attenuates onset and progression of the disease [100]. Interestingly, MALT1-PD mice are completely protected from induction of EAE [10, 11], suggesting an important role of MALT1 in the generation and activation of autoreactive T cells. TH17 cells play a major role in the pathology of EAE, and both MALT1-deficient and MALT1-PD mice show an impaired differentiation of naïve T cells into TH17 cells in vitro [11, 14]. Interestingly, MOG-immunized MALT1-PD mice also have reduced IL-17 positive T cells in the CNS [10], suggesting that MALT1 protease-mediated signals are required for the differentiation of autoreactive TH17 cells.

In a second autoimmune setting, using a T cell transfer model of colitis, we demonstrated that naïve T cells from these mice less efficiently induce colon inflammation than wild-type T-cells [10]. Unfortunately, the analysis of other models of autoimmune diseases in MALT1-PD mice has been severely hampered by the fact that these mice spontaneously develop a severe form of autoimmune disease (Table 1). Indeed, MALT1-PD mice fail to thrive, show neurological defects, but most intriguingly, exhibit a very pronounced lymphadenopathy with a strong increase in B- and T cell numbers in the lymph nodes, the presence of T cells with an effector/memory phenotype and lymphocyte infiltration into various organs, including the stomach, glands, lungs, peripheral nerves and other organs [10–13]. Similar to MALT1-deficient mice, MALT1-PD mice have a defect in regulatory T cell development, which leads to strongly reduced regulatory T cell numbers in the thymus, spleen and lymph nodes [10–12]. In contrast to T cells from MALT1 knock-out mice, which are refractory to stimulation, T cells from MALT1-PD mice retain some residual activation potential, most likely though MALT1 scaffold-dependent IKK and JNK activation [10–13]. The imbalance between the residual partial T cell activation potential on the one hand, and the lack of efficient counter-regulation by regulatory T cells on the other hand, most likely leads to the observed fatal autoimmune disease in the otherwise immune-compromised animals. Interestingly, some patients with MALT1 mutations also show combined signs of immunodeficiency and autoimmune features (such as inflammatory bowel disease) that correlate with reduced peripheral Treg numbers [16, 17].

**Table 1:** Features of MALT1 protease-dead mice, summarizing defects in lymphocyte development and leukocyte activation and responses to disease models

Observed features	References
**Lymphocyte development**	
Reduced number of regulatory T cells	[10–12]
Impaired development of B1 and MZ B cells	[10–13]
Increased CD3 expression on DN4 thymocytes	[10]
**Leukocyte properties**	
Impaired T cell activation	[10, 11, 13]
Impaired NK cell activation	[10]
Impaired DC activation	[10, 13]
Impaired B cell response to immunization	[10, 11, 13]
**Histological findings/pathology**	
Failure to thrive, weight loss	[10–13]
Neurological disorder and paralysis	[11–13]
Loss of Purkinje cells in cerebellum, elevated T cell numbers in the brain	[12]
Increased percentages of effector/memory T cells	[10–12]
Increased percentages of CD4^+^ T cells expressing IFNγ and IL-4	[10, 11]
Elevated serum concentrations of IFNγ and TNFα	[12]
Lymphadenopathy with strong increase in T and B cell numbers	[10–12]
Lymphoid cell infiltration in glandular stomach, glands, lungs, peripheral nerves and other organs	[10–13]
Increased serum levels of IgG1 and IgE	[10, 11]
Autoantibodies recognizing parietal cells of the stomach	[10]
**Response in experimental disease models**	
Protection from experimental autoimmune encephalomyelitis (EAE)	[10, 11]
Lower penetrance of T cell transfer colitis	[10]

The compromised Treg function in MALT1-deficient or -mutant humans and mice raises the concern that prolonged treatment of individuals with MALT1 inhibitors may favor the development of autoimmunity. It is important to note, however, that the in vitro differentiation of naïve T cells from MALT1 PD mice into regulatory T cells was possible [10, 11], indicating that this process would not be severely hampered by a therapeutic MALT1 inhibitor. Along these lines, mepazine did not change the percentages of regulatory T cells in mice that were immunized with MOG peptide and subsequently treated daily with the inhibitor for up to 17 days [100]. Furthermore, the presence of mepazine did not impede the in vitro polarization of naïve mouse T cells into regulatory T cells [100]. Thus, MALT1 is strictly required for the development of natural Treg cells, while MALT1-independent mechanisms seem to be important for the generation of inducible Treg cells. Further experiments are however required to assess potential adverse effects of MALT1 inhibition on Treg cell development and function.

## Conclusions and perspectives

MALT1 has an essential role in the immune response and the growth of lymphoma cells with constitutive MALT1 activity. Studies with MALT1-deficient mice and MALT1 inhibitors have suggested that MALT1 could be a rational drug target for immunomodulation and lymphoma treatment. The recent development of mice expressing catalytically inactive MALT1 confirms not only its essential role in the immune response, but also highlights the fact that MALT1 activity is required for the development of natural Treg cells that prevent autoimmunity. Importantly, MALT1 inhibition does not prevent the differentiation of naïve T cells into Treg cells. Additional studies are thus required to assess whether the long-term use of MALT1 inhibitors may affect Treg functions in the adult, which is of obvious concern for immunomodulation, but maybe less evident for the treatment of lymphomas.

An important perspective for future research on MALT1 will be to further explore its molecular function and to identify the full spectrum of its substrates, which may open additional windows for therapeutic applications. Another major challenge will be to better understand MALT1’s physiological function in non-immune cells, and its relevance not only for immune but also for non-immune pathologies.

## Abbreviations

ABC: Activated B cell
BCL10: B-cell lymphoma-10
CARD: Caspase recruitment domain
CARMA1: CARD-containing membrane-associated guanylate kinase-1
DLBCL: Diffuse large B-cell lymphoma
EAE: Experimental autoimmune encephalomyelitis
GCB: Germinal center B cell
MALT1: Mucosa-associated lymphoid tissue protein-1
MCL: Mantle cell lymphoma
